# Predicting the immune therapy response of advanced non-small cell lung cancer based on primary tumor and lymph node radiomics features

**DOI:** 10.3389/fmed.2025.1541376

**Published:** 2025-04-03

**Authors:** Dong Xie, Jinna Yu, Cong He, Han Jiang, Yonggang Qiu, Linfeng Fu, Lingting Kong, Hongwei Xu

**Affiliations:** ^1^Department of Radiology, Shaoxing Second Hospital Medical Community General Hospital, Shaoxing, China; ^2^Department of Medical Oncology, Shaoxing Second Hospital Medical Community General Hospital, Shaoxing, China

**Keywords:** non-small cell lung cancer, lymph nodes, radiomics, delta, immunity, prediction model

## Abstract

**Objective:**

To identify imaging biomarkers of primary tumors and lymph nodes in patients with stage III–IV non-small cell lung cancer (NSCLC) and assess their predictive ability for treatment response (response vs. non-response) to immune checkpoint inhibitors (ICIs) after 6 months.

**Methods:**

Retrospective analysis of 83 NSCLC patients treated with ICIs. Quantitative imaging features of the maximum primary lung tumors and lymph nodes on contrast-enhanced CT imaging were extracted at baseline (time point 0, TP0) and after 2–3 cycles of immunotherapy (time point 1, TP1). Delta-radiomics features (delta-RFs) were defined as the net changes in radiomics features (RFs) between TP0 and TP1. Interobserver interclass coefficient (ICC) and Pearson correlation analyses were applied for feature selection, and logistic regression (LR) was used to build a model for predicting treatment response.

**Results:**

Four and five important delta-RFs were selected to construct the nodal and tumor models, respectively. Δ Tumor diameter was used for constructing the clinical prediction model. The predictive efficacy of the nodal model for the treatment response status was higher than that of the tumor and clinical models. In the training set, the AUC values for the three models were 0.96 (95% CI = 0.90–1.00), 0.86 (95% CI = 0.76–0.95), and 0.82 (95% CI = 0.71–0.93), respectively. In the validation set, the AUC values were 0.94 (95% CI = 0.85–1.00), 0.77 (95% CI = 0.56–0.98), and 0.74 (95% CI = 0.48–1.00), respectively.

**Conclusion:**

The nodal model based on delta-RFs performed well in distinguishing responders from non-responders and could identify patients more likely to benefit from immunotherapy. Finally, the nodal model exhibited a higher classification performance than the tumor model.

## 1 Introduction

Non-small cell lung cancer (NSCLC) accounts for 85% of all lung cancers and is the leading cause of cancer-related deaths worldwide (1). Despite recent advancements in lung cancer treatment, the 5-year survival rate of patients with lung cancer remains disappointing at only 15% (2). In recent years, immune checkpoint inhibitors (ICIs) have improved the treatment outcomes of patients with advanced NSCLC without targetable mutations. However, according to published evidence (3), the increase in progression-free survival (PFS) and/or overall survival (OS) is still limited to a small percentage of patients (15–30%). Although the expression of the tumor cell PD-L1 has been widely used as a biomarker for selecting patients for immune therapy (2, 4), the relationship between PD-L1 expression and the efficacy of ICIs treatment remains uncertain.

Radiomics is an emerging field in medical imaging, which can quantify medical imaging data and translate qualitative clinical problems into quantitative ones, thus providing a more objective approach to solving clinical problems (5). Recent studies (6) have shown that non-invasive diagnostic images can describe the phenotype of lung tumors, and their use could be feasible to predict the survival stratification of patients with advanced NSCLC under different treatment methods. In these noninvasive imaging-based prediction or classification models, a radiomics method based on CT images has been developed and applied to establish prognosis prediction models, evaluate the effectiveness and necessity of different treatment methods, and predict early clinical outcomes. More specifically, traditional radiomics methods use baseline medical images for evaluation or prediction and ignore changes in tumors during treatment or follow-up. Alternatively, delta radiomics utilize changes in radiomic features (RFs) during or after treatment to guide clinical decision-making and may be more suitable for evaluating tumor responses to treatment (7, 8).

In locally advanced NSCLC, tumors tend to spread from the primary site to lymph nodes. Pretreatment lymph node staging is closely associated with disease progression and poor prognosis (9). As such, involved lymph nodes may have unique phenotypic characteristics related to the biological processes that affect disease spread, and thus, treatment response. In this study, we hypothesized that the presence of more invasive cancer cells in the metastatic mediastinal/paratracheal lymph nodes may determine prognosis and provide additional valuable information regarding the primary tumors of patients with NSCLC. To prove this, we analyzed the delta-radiomic features (delta-RFs) of the primary tumor and metastatic lymph nodes based on contrast-enhanced CT (CE-CT) scans and further validated these results in an independent cohort.

## 2 Materials and methods

### 2.1 Patients

This was a retrospective analysis of patients with NSCLC treated with ICIs at Shaoxing Second Hospital between January 2016 and November 2022. Tumor staging was performed according to the 8th edition of the American Cancer Joint Committee TNM staging criteria (10). All patients were pathologically diagnosed with III–IV NSCLC. This study adhered to the principles of the Declaration of Helsinki and was approved by the hospital’s Ethics Committee [Approval No. Ethics Approval (2022018)].

The inclusion criteria were as follows: (1) NSCLC confirmed by histology, (2) first- or later-line treatment with ICIs, and (3) complete baseline demographic data before treatment. The exclusion criteria were as follows: (1) baseline imaging (time point 0, TP0) or follow-up after 2–3 cycles of immunotherapy (time point 1, TP1) without CE-CT, (2) inability to accurately evaluate lesion boundaries on CE-CT images, (3) time interval between baseline imaging and immunotherapy exceeds 4 weeks; and (4) short axis of lymph nodes less than 15 mm.

### 2.2 CT image acquisition

A 64-slice CT scanner (Siemens SOMATOM Definition AS, Germany) was used to examine the patients who underwent routine respiration training. The scanning parameters were as follows: tube voltage = 120 kV, tube current = 200–300 mA, rotation time = 0.75 s, collimation = 32×1.25 mm, FOV = 360.0–500.0 mm, matrix = 512 × 512, slice thickness and interval = 5.0 mm, contrast agent injection rate = 2.5–3.0 mL/s, and injection volume = 1.1–1.7 mL/kg. After the routine scan, a thin-layer post-processing reconstruction of 0.6–1.5 mm was performed.

### 2.3 Image analysis

Two radiologists (A and B) with 15 years of experience in chest radiography independently evaluated the images, and the final results were obtained through consultation in cases of disagreement. The observed indicators included the selection of target lesions, target lesion boundaries, and TNM staging. This study evaluated whether the target lesions progressed after 6 months of treatment, based on the Response Evaluation Criteria in Solid Tumors (RECIST, version 1.1) (11).

### 2.4 Target lesion segmentation

**Figure 1:**
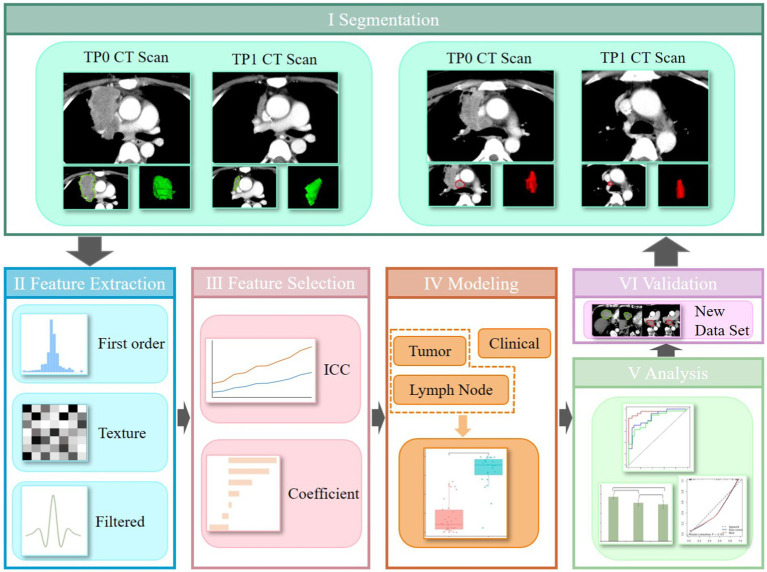
The workflow of this study, which mainly composed of six steps: data set, feature extraction, feature selection, model building, analysis, and validation.

ITK-SNAP software (version 3.6.0, http://www.itksnap.org/) was used for tumor segmentation. Radiologist A manually delineated the region of interest (ROI) for lesions on TP0 and TP1 CE-CT images, as shown in Figure 1I. To ensure reproducibility and accuracy, the radiologists separately segmented the lesion ROIs and extracted the features at TP0 and TP1 from 10 randomly selected patients. Interobserver interclass coefficient (ICC) was used to determine the consistency of these features, with an ICC value greater than 0.75 indicating higher repeatability of the results.

### 2.5 Image preprocessing and RFs extraction

The PyRadiomics package was used to analyze the segmentation data and isolate phenotypic features from the tumor regions after manual segmentation. To standardize all voxel sizes among the patients, the CT images were resampled to a 2-mm resolution in all three directions (Figure 1II). To avoid redundancy with traditional radiological features and highlight texture differences within the target lesions, shape features were excluded from the RFs extraction. A total of 1,050 RFs were extracted for each 3D ROI, including the first-order features, grey-level co-occurrence matrix (GLCM), grey-level run length matrix (GLRLM), grey-level size zone matrix (GLSZM), grey-level dependence matrix (GLDM), and neighborhood grey-tone difference matrix (NGTDM). Delta-RFs were defined as the net change in RFs extracted at TP0 and TP1: Delta-RFs = Feature (TP1) − Feature (TP0). All features were normalized using the *Z*-score in Excel.

### 2.6 Delta-RFs and clinical features selection

#### 2.6.1 Delta-RFs selection

A five-fold cross-validation method was used to assign training and validation cohorts using the same random seed in all splits to ensure consistency in grouping. Feature selection was performed using ICC and correlation analyses (Figure 1III). First, features with inter-observer instability (ICC <0.75) were excluded. Features with a high correlation (Pearson’s correlation coefficient >0.9) were eliminated. The most significant predictive features and their corresponding weight coefficients were selected and the radiomics score (Rad score) for each patient was calculated to build the radiomics model.

#### 2.6.2 Clinical features selection

Clinical feature selection involved statistical tests, multivariate analyses, and stepwise regression to select features associated with the six-month treatment response. These features were used to develop the clinical model.

### 2.7 Construction of radiomics prediction model

Significant radiomics and clinical features were selected as independent variables, while the six-month treatment response was the dependent variable. Logistic regression (LR) was used to establish a multivariate regression model to predict the treatment response (Figure 1IV). Nodal, tumor, and clinical models were constructed, and the receiver operating characteristic (ROC) curve and area under the curve (AUC) were used to evaluate the predictive performance of the rad-score for immunotherapy efficacy in advanced lung cancer.

### 2.8 Model comparison and evaluation

The performance of the three classifiers was comprehensively evaluated using ROC curves, AUC, accuracy (ACC), sensitivity (SEN), specificity (SPE), negative predictive value (NPV), and positive predictive value (PPV) (Figure 1V). The DeLong’s test was used to compare the ROC curves of the three models. Calibration curves were used to describe the predictive accuracy of the three models. All the models were validated using a validation cohort (Figure 1VI).

### 2.9 Statistical methods

Data analysis was performed using SPSS (version 26.0) and R (version 4.1.2; https://www.r-project.org/). Continuous data are presented as mean ± standard deviation ($\bar{x\;}\pm s$) and were analyzed using independent sample *t*-tests. Categorical data are presented as percentages [*n* (%)] and were analyzed using chi-square or Fisher’s exact tests. Univariate and multivariate LR analyses were conducted, and the backward stepwise variable elimination method was used to select clinically significant features to build the clinical prediction model. Statistical significance was set at *p* < 0.05.

## 3 Results

### 3.1 Population demographics

A total of 83 NSCLC patients were included in this study, including 69 (83.1%) males and 14 (16.9%) females, with an age range of 36 to 85 years, and a median age of 67 years. The demographic and clinicopathological characteristics of the 83 patients are presented in Table 1. Among them, 48 (57.8%) were in the responder group and 35 (42.2%) were in the non-responder group. Fifty-three (54.6%) of all patients received PD-1 ICIs (camrelizumab, sintilimab, tislelizumab or nivolumab) or PD-L1 ICIs (atezolizumab) monotherapy. The remaining 44 (45.4%) patients were treated with the combination of immunotherapies, ICIs in combination with chemotherapeutic agents (gemcitabine + cisplatin, paclitaxel + carboplatin) and/or antiangiogenic agents (mainly bevacizumab, endo, anlotinib, and afatinib). However, there were statistically significant differences in tumor diameter and lymph node diameter between the responder and non-responder groups (*p* < 0.05).

**Table 1:** Baseline data of the responders and non-responders.

Demographic or clinicopathologic characteristic	Responders (*N* = 48)	Non-responders (*N* = 35)	*p*-value
Gender, No. (%)			0.213
Female	6 (12.5%)	8 (22.9%)	
Male	42 (87.5%)	27 (77.1%)	
Age, [M (Q1, Q3)]	67 (59, 73)	67 (62, 71)	0.969
Tobacco use, No. (%)			0.564
Never smoker	24 (50.0%)	16 (45.7%)	
Current smoker	15 (31.3%)	9 (25.7%)	
Former smoker	9 (18.8%)	10 (28.6%)	
Pathological type, No. (%)			0.329
Squamous cell	35 (72.9%)	22 (62.1%)	
Adenocarcinoma	13 (27.1%)	13 (37.1%)	
Pathologic N stage, No. (%)			0.238
N1	2 (4.2%)	1 (2.9%)	
N2	22 (45.8%)	10 (28.6%)	
N3	24 (50.0%)	24 (68.6%)	
TNM stage, No. (%)			0.622
III	19 (39.6%)	12 (34.3%)	
IV	29 (60.4%)	23 (65.7%)	
Line of treatment, No. (%)			0.098
First line	32 (66.7%)	17 (48.6%)	
Later line	16 (33.3%)	18 (51.4%)	
Treatment strategy, No. (%)			0.309
Monotherapy	22 (45.8%)	20 (57.1%)	
Combination therapy	26 (54.2%)	15 (42.9%)	
Tumor diameter at pre-treatment (mm)	55.3 ± 19.7	58.7 ± 22.1	0.540
Lymph nodal diameter at pre-treatment (mm)	19.5 ± 6.2	20.4 ± 7.0	0.534
Δ Tumor diameter (mm)	−13.4 ± 9.5	0.3 ± 14.1	0.000^**^
Δ Lymph nodal diameter (mm)	−5.8 ± 5.6	1.6 ± 7.4	0.000^**^

^**^p < 0.001.

### 3.2 Delta-RFs selection and model construction

**Figure 2:**
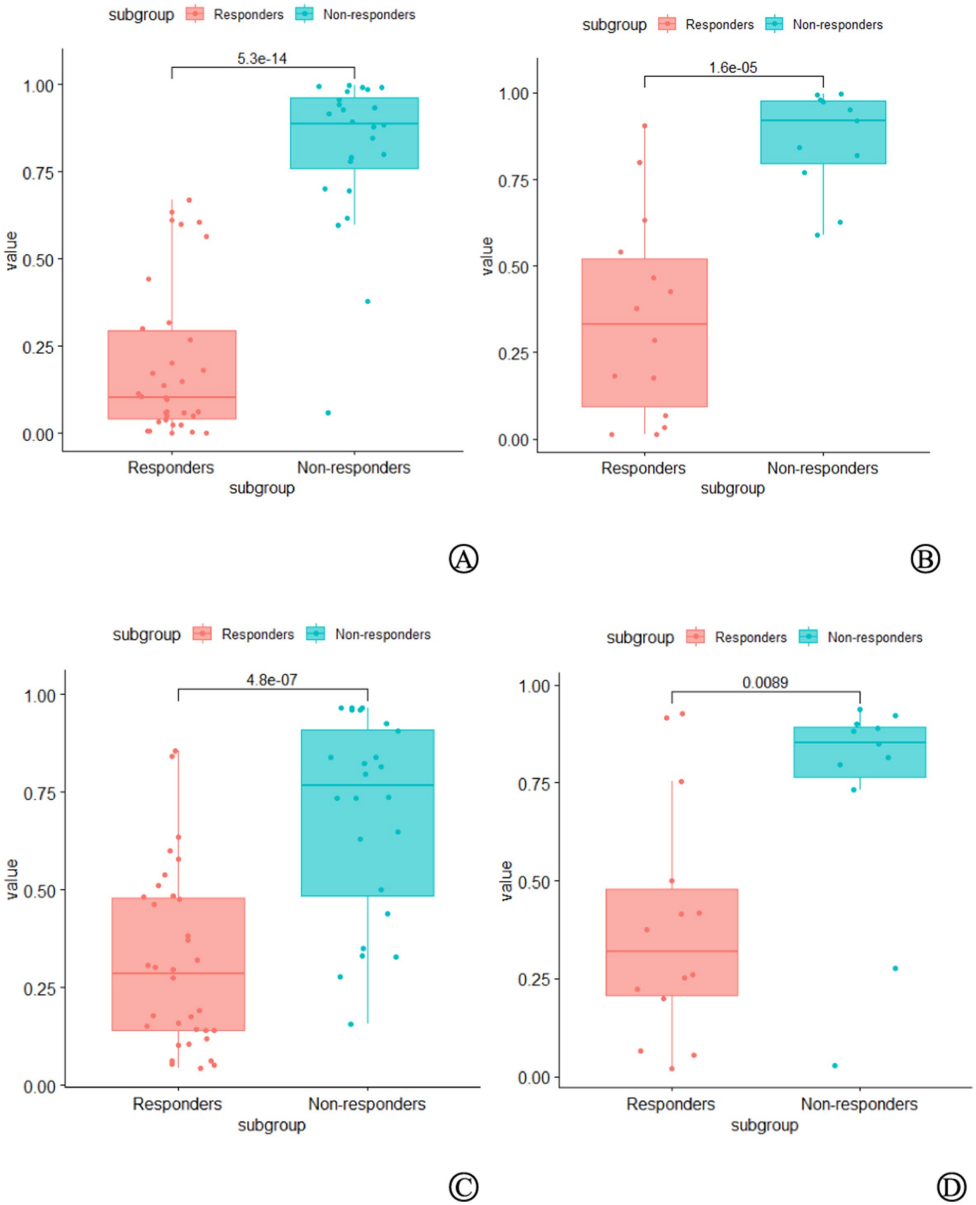
Comparison of delta radiomics scores between the non-responders group and the responders group in the training set **(A)** and validation set **(B)** of the nodal model, as well as in the training set **(C)** and validation set **(D)** of the tumor model. In both groups, the delta radiomics scores of the non-responders group were significantly higher than those of the responders group (*p* < 0.05).

A total of 1,050 delta-RFs were extracted, and four key RFs (nodal model) and five key RFs (tumor model) were selected after ICC and correlation analysis, as shown in Table 2. Based on these features, the LR algorithm was applied to train the delta-RF sets of each lymph node and primary tumor, construct the nodal and tumor models, and convert the output probability scores into delta rad-scores. There were significant differences in the delta rad-scores between the responder and non-responder groups (Figure 2).

**Table 2:** Results of logistic regression analysis of screened radiomics features.

Models	Screened radiomics features	Odds ratio	OR 95%CI	*p*-value
Node model	wavelet-LLL_glszm_ZoneEntropy	11.113	3.293–37.506	0.000^**^
	log-sigma-4-0-mm-3D_glszm_ZoneEntropy	10.388	2.865–37.673	0.000^**^
	log-sigma-4-0-mm-3D_glszm_GrayLevelNonUniformity	1.706	1.265–2.300	0.000^**^
	log-sigma-3-0-mm-3D_glrlm_RunLengthNonUniformity	1.022	1.010–1.035	0.000^**^
Tumor model	log-sigma-2-0-mm-3D_glszm_ZoneEntropy	6.507	2.286–18.525	0.000 ^**^
	log-sigma-3-0-mm-3D_glrlm_RunEntropy	5.153	1.953–13.597	0.001^*^
	log-sigma-4-0-mm-3D_glrlm_LongRunHighGrayLevelEmphasis	1.015	1.007–1.023	0.000^**^
	log-sigma-4-0-mm-3D_glrlm_LongRunEmphasis	1.023	1.010–1.036	0.001^*^
	wavelet-LLL_gldm_DependenceNonUniformityNormalized	3.489	1.608–7.570	0.002^*^

^*^p < 0.05 and ^**^p < 0.01. HR, hazard ratio; CI, confidence interval.

### 3.3 Clinical prediction model establishment

The results of the multivariate LR analysis showed that Δ Tumor diameter was an independent prognostic factor affecting the efficacy of ICIs treatment in patients with NSCLC (*p* < 0.05), as shown in Table 3. A clinical prediction model (clinical model) was established based on the selected independent variables.

**Table 3:** Results of logistic regression analysis of clinical variables in the training set.

Demographic or clinicopathologic characteristic	Odds ratio	OR 95% CI	*p*-value
Gender (male vs. female)	8.900	0.634–124.867	0.105
Age	1.026	0.937–1.125	0.577
Tobacco use never smoker vs. current smoker	0.464	0.051–4.205	0.495
Former smoker	1.636	0.239–11.189	0.616
Pathological type (squamous cell vs. adenocarcinoma)	1.716	0.286–10.313	0.555
Pathologic N stage N1 vs. N2	0.483	0.014–16.939	0.688
N3	2.925	0.114–75.353	0.517
TNM stage (III vs. IV)	0.466	0.059–3.670	0.469
Line of treatment (first line vs. later line)	0.726	0.095–5.581	0.759
Tumor diameter at pre-treatment	1.042	0.999–1.087	0.055
Lymph node diameter at pre-treatment	1.033	0.923–1.156	0.571
Δ Tumor diameter	0.878	0.794–0.970	0.011
Δ Lymph node diameter	0.879	0.765–1.010	0.070

CI, confidence interval.

### 3.4 The performance of the nodal model, tumor model, and clinical model

**Figure 3:**
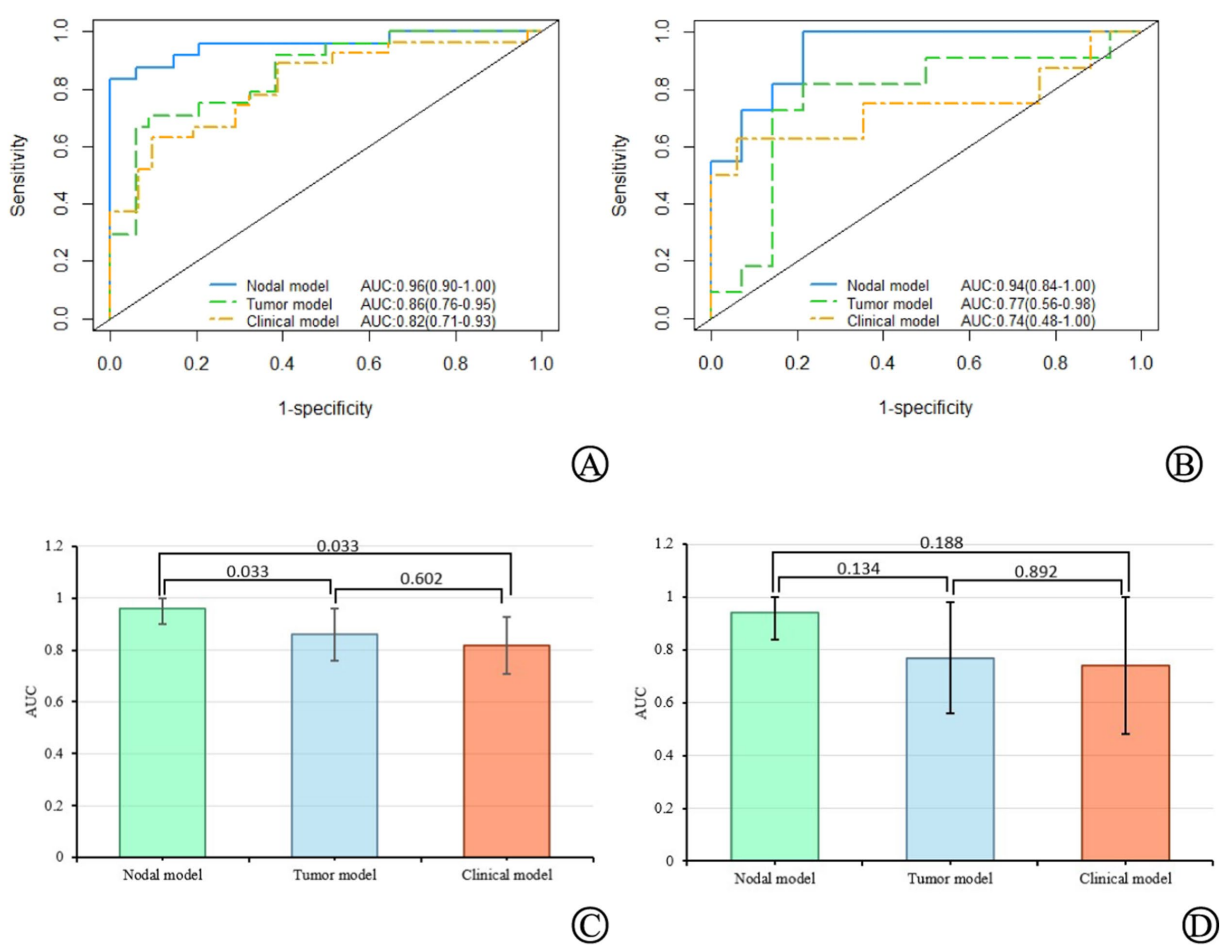
The AUC values of the nodal, tumor, and clinical models, as well as their comparisons in the training set **(A,C)** and validation set **(B,D)**.

In the training set, the nodal model had the highest AUC of 0.96 (95% CI = 0.90–1.00), significantly higher than the tumor (0.86, 95% CI = 0.76–0.95) (DeLong test, *p* < 0.05) and clinical models (0.82, 95% CI = 0.71–0.93) (DeLong test, *p* < 0.05). The AUC of the tumor model was higher than that of the clinical model (DeLong’s test, *p* > 0.05). In the validation set, the AUC of the nodal model (0.94, 95% CI = 0.85–1.00) was higher than the tumor model (0.77, 95% CI = 0.56–0.98) and the clinical model (0.74, 95% CI = 0.48–1.00), but the differences were not statistically significant (DeLong test, *p* > 0.05). Finally, the AUC of the tumor model was higher than that of the clinical model (DeLong’s test, *p* > 0.05) (Figure 3). The calibration curve along with the Hosmer–Lemeshow test (*p* > 0.05) demonstrated good consistency between the predictions and observations in the three models (Supplementary Figure 1).

Using the Youden index to determine the cutoff value of the ROC curve, the ACC, SEN, SPE, NPV, and PPV of the nodal, tumor, and clinical models were calculated. The results are summarized in Table 4. Except for SPE and PPV, which were the highest in the clinical model, all other indicators were the highest in the nodal model.

**Table 4:** Performance metrics of the models.

Model	Training set						Validation set					
	AUC	ACC	SEN	SPE	NPV	PPV	AUC	ACC	SEN	SPE	NPV	PPV
Nodal	0.96	0.86	0.92	0.82	0.93	0.79	0.94	0.84	1.00	0.86	1.00	0.82
Tumor	0.86	0.78	0.75	0.79	0.82	0.72	0.77	0.80	0.82	0.79	0.85	0.75
Clinical	0.82	0.69	0.67	0.71	0.71	0.67	0.74	0.84	0.50	1.00	0.81	1.00

## 4 Discussion

The data of the SEER (surveillance, epidemiology, and end results) database in the United States in 2018 showed that the overall 5-year survival rate of lung cancer patients was 18.6%, while the 5-year survival rate of advanced lung cancer patients with distant metastases was only 4.7%. With the advent of ICIs, the 5-year survival rate of patients with advanced lung cancer increased to 16% for the first time (12), which became a breakthrough in the treatment of advanced NSCLC. The ICI response of NSCLC patients varies widely among individuals, and the prognosis is influenced by a variety of factors, so it is necessary to find reliable biomarkers to screen the population that may benefit from ICIs (13).

Despite the availability of new treatment methods, patient survival rates remain relatively low (14). Although many clinical and histopathological features, laboratory markers, molecular biomarkers, and genetic markers have been tested for potential prognostic value, few effective and accurate prognostic factors are currently used in clinical practice to manage or predict individual patient prognosis. Clinical trials (15) have shown that lymph node clearance is closely related to patients’ overall survival. Lymph nodes are common sites of regional metastases and are crucial for cancer staging. Lymph node phenotypic characteristics contain valuable information that is particularly relevant to patients with advanced NSCLC and can effectively predict clinical endpoints. There is also evidence that lymph node imaging features have a higher prognostic value than primary tumors in patients with lung, head, and neck cancers (16, 17). However, despite extensive research investigating the relationship between primary tumor phenotypes and clinical outcomes, there has been little quantitative analysis of the correlation between NSCLC lymph node characteristics and clinical outcomes. Coroller et al. (17) conducted the first study using quantitative lymph node imaging features to predict tumor response to radiochemotherapy. Carvalho et al. (18) found that common standard uptake value (SUV) descriptors from metastatic lymph nodes were associated with overall patient survival in NSCLC. Furthermore, compared to positron emission tomography (PET) information extracted solely from primary tumors, PET information extracted from metastatic lymph nodes had a higher prognostic value (*C*-index: 0.62 vs. 0.53, 0.56 vs. 0.54, respectively). Our study is based on the fundamental principle that disease progression and metastatic ability are closely related to the presence of metastatic lymph nodes. We extracted quantitative features related to immunotherapy response from CE-CT images of both primary tumors and affected lymph nodes. We found that the predictive efficacy of the nodal model was superior to that of the tumor model (AUC: 0.96 vs. 0.86, 0.94 vs. 0.77, respectively). Although there was no statistically significant difference in the validation group, the tumor model had a lower 95% CI limit of only 0.56 with a wide interval, indicating that the predictive efficacy of the tumor model was relatively low. The clinical model also exhibited a similarly poor performance, confirming that lymph node radiomic information based on CE-CT scans provides additional prognostic information to that obtained from primary tumors (19). Coroller et al. (17) demonstrated that the optimal radiomics features extracted from metastatic lymph nodes can predict the pathological response after radiochemotherapy in patients with NSCLC, and this performance is higher than that of the optimal radiomics features extracted from primary tumors (AUC: 0.75 vs. 0.61, *p* = 0.03), further confirming the point above.

Moreover, previous radiomics studies have mainly focused on analyzing pretreatment imaging features. In our study, we provided a more comprehensive description of the rich temporal dependence between primary tumors and lymph nodes in pre-treatment and mid-treatment scans. This information can provide insights into treatment-induced changes, dynamically evaluate tumor burden, and better align with the evaluation of immunotherapy efficacy in clinical practice, which is consistent with recent reports (20, 21). In a similar study, Liu et al. (20) extracted delta-RFs from primary lesions and mediastinal metastatic lymph nodes in patients with late-stage NSCLC to predict the response status to ICIs treatment after 6 months. They found that the predictive performance of delta-RFs (AUC: 0.80–0.82) was significantly higher than that of baseline radiomics features (AUC: 0.51–0.59). In the present study, the predictive performance of the delta radiomics model reached a maximum of 0.96. Delta radiomics has been proposed to evaluate the changes that occurred during treatment after time by accessing changes in RFs of different timeline CT scans. Delta-radiomics has greater reproducibility and stability than conventional imaging histology (22). In addition to the fact that delta-RFs have been shown to be effective in differentiating responders from non-responders in advanced non-small cell lung cancer undergoing immunotherapy, delta-RFs have also shown good efficacy in treatment response assessment in patients with metastatic melanoma (23). In a recent study, Fan et al. (24) for the first time assessed tumor response in patients with esophageal squamous cell carcinoma undergoing neoadjuvant chemoradiotherapy based on delta-RFs of CT images. The model based on delta-RFs had higher predictive power than previous studies, especially when combined with clinical factors, further improving the predictive performance with an AUC of 0.963. In the clinical model, Δ Tumor diameter was an independent risk factor for prognosis. This represents the change in the diameter of the primary tumor caused by treatment, indicating that mid-treatment scans can provide important information related to clinical outcomes, supplementing the information provided by pretreatment imaging features.

Owing to the large number of features included in radiomics, we used the ICC and Pearson correlation analysis to select the most critical delta-RFs. Four and five optimal features were selected to construct the nodal and tumor models, respectively. Interestingly, we found that the zone entropy (ZE) of the GLSZM was the highest-weighted delta-RF regardless of the nodal or tumor model. The ZE measures the uncertainty/randomness in the distribution of zone sizes and grey levels, with a higher value indicating greater heterogeneity in the texture patterns. In our cohort, the ZE values were significantly higher in the non-responder group than in the responder group, both in the nodal model and the tumor model. The level of the ZE value may reflect the heterogeneity of the target lesions, with higher ZE values indicating higher heterogeneity of the target lesions, which is more likely to cause drug resistance.

Our study had certain limitations. First, this was a retrospective study based on a single medical center, and our model lacked external validation. Studies have shown that variations in scanning devices, acquisition methods, reconstruction parameters, and scanning protocols may affect the subsequent feature analysis (25, 26). Therefore, we believe that designing prospective trials and standardizing imaging scans for all patients from different research institutions is necessary. Secondly, the sample size of our cohort was relatively small, and the robustness and effectiveness of the model must be validated using larger datasets. Nevertheless, our study included a larger dataset (83 patients) than previous studies on lymph nodes (27, 28) (which included 25 and 43 patients). Third, limited follow-up was conducted in some patients; therefore, PFS and OS analyses were not performed on this dataset. However, due to the advanced stage of the tumors, our follow-up period was sufficient to provide clinically relevant information. Fourth, the included lymph nodes in this study were not confirmed by pathology but were included based on the diagnostic criteria of RECIST 1.1 for pathological lymph nodes (short axis greater than 15 mm). The diagnosis of mediastinal/hilar lymph node metastasis is usually performed through ^18^F-fluorodeoxyglucose positron-emission tomography/computed tomography (^18^F-FDG PET/CT), endobronchial ultrasound/endoscopic ultrasound (EBUS/EUS), or mediastinoscopy (29). However, ^18^F-FDG PET/CT has significant limitations, particularly regarding the uptake of glucose-like FDG in benign inflammatory lymph nodes, which may lead to false-positive results (30). In addition, the low spatial resolution of PET/CT hinders the detection of small metastatic lymph nodes. Invasive examinations are often constrained by the anatomy and are only limited to nodes accessible through this approach. CE-CT can help reduce the number of invasive surgeries required to confirm lymph node metastasis, thereby reducing the complications associated with invasive procedures. Therefore, CE-CT has a wider potential for clinical applications, does not require additional ionizing radiation, and does not incur significant additional costs. Fifth, in the clinical model of this study, wide confidence intervals were observed. Due to the small sample size, when using 5-fold cross-validation to evaluate model performance, the limited sample size in each fold may not fully represent the overall data, which could affect the stability of the model’s performance. In future research, we aim to explore other methods, such as bootstrapping, to increase the diversity of the sample size through repeated sampling, thus mitigating the fluctuations caused by the small sample size.

In conclusion, this study demonstrated that lymph node-based phenotypic features are superior in reflecting the potential sensitivity of patients to immunotherapy than primary tumor sites. This allows for the early detection of patients with a high likelihood of rapid progression to ICIs treatment. Predicting the tumor response early during immunotherapy has potentially significant clinical implications for precision medicine. If the model predicts a poor response to immunotherapy, clinicians can consider pausing or switching the treatment plan, thereby avoiding continued use of potentially ineffective therapy, saving treatment resources, and minimizing side effects. Additionally, the model can help identify patients who require treatment adjustments, such as through combination therapy or changing the immunotherapy approach. Based on the early response predicted by the model, clinicians can develop a personalized management plan for the patient. This may include enhanced monitoring, regular evaluations, and treatment adjustments to ensure the optimal therapeutic outcome.

## Data Availability

The original contributions presented in the study are included in the article/Supplementary material, further inquiries can be directed to the corresponding author.
