# Supplementary material for: Predicting the immune therapy response of advanced non-small cell lung cancer based on primary tumor and lymph node radiomics features
Source: Front Med (Lausanne). 2025 Apr 3;12:1541376. doi: 10.3389/fmed.2025.1541376 (PMC12003267; doi:10.3389/fmed.2025.1541376)
Supplement: Supplementary file 1 [file Supplementary_file_1.docx]

Supplementary file 1

# Supplementary Figures


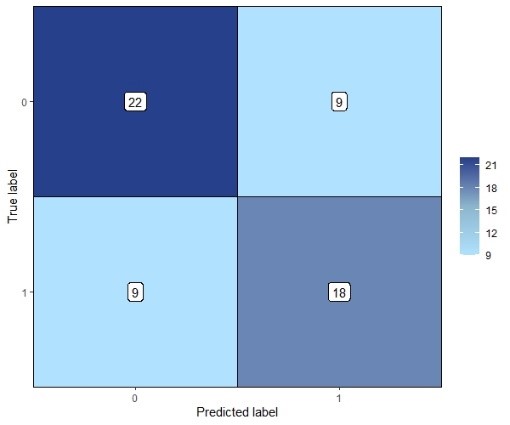

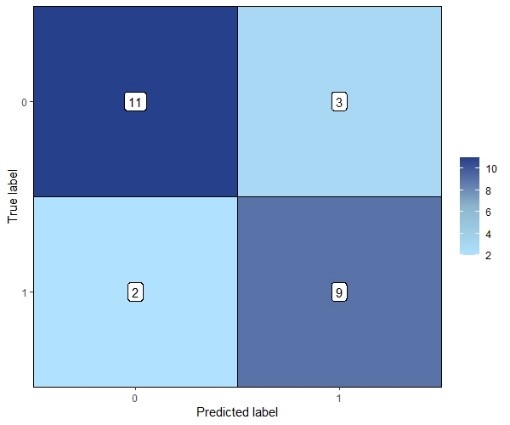


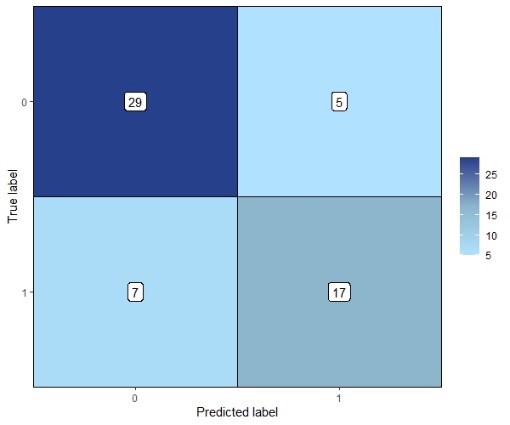

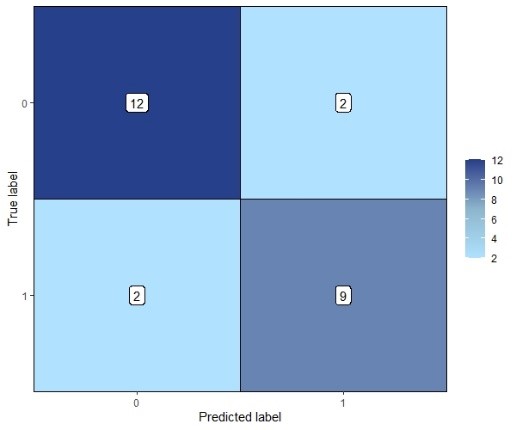


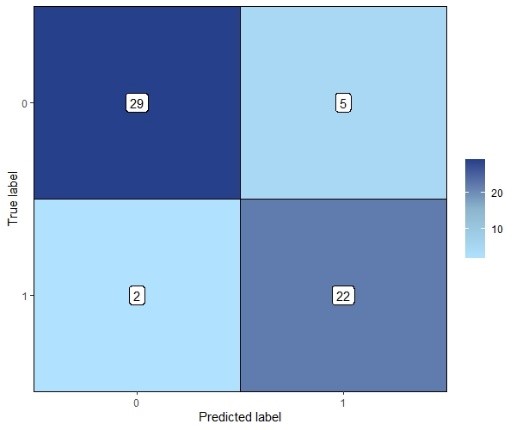

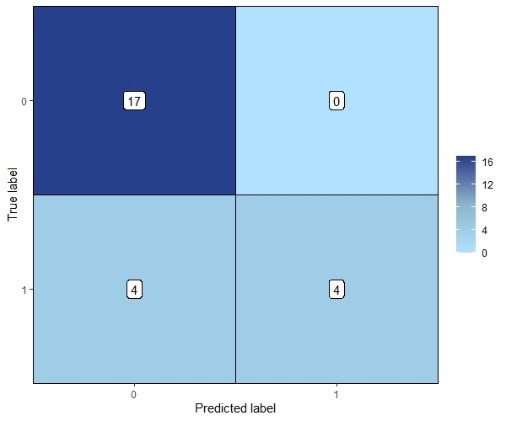


**Supplementary Figure 1.** Confusion matrixes of the three models. Training set: Nodal model(A), Tumor model(C), Clinical model(E); Validation set: Nodal model(B), Tumor model(D), Clinical model(F).
